# A null allele in the *wdfy-3* selective autophagy gene of *C. elegans* .

**DOI:** 10.17912/micropub.biology.000598

**Published:** 2022-07-20

**Authors:** Tyler Buddell, Christopher Quinn

**Affiliations:** 1 University of Wisconsin-Milwaukee, Milwaukee, WI, USA

## Abstract

The *C. elegans* WDFY-3 protein is important for cargo selection during selective autophagy and for regulating axon termination. The C-terminal region of WDFY-3 contains BEACH, WD repeats, and FYVE-like domains, all of which are required for selective autophagy. WDFY-3 also contains a large N-terminal region that is relatively uncharacterized. Currently, *wdfy-3(ok912)* is the only mutant allele that has been characterized for this gene. This allele features a small deletion that is predicted to disrupt the C-terminal region of the protein. Here, we used CRISPR Cas9 to produce a new *wdfy-3(cue30)* allele that is a near complete deletion of the coding region. We report that, unlike the existing *wdfy-3(ok912)* allele, this new *wdfy-3(cue30)* null allele causes a weak overextension phenotype in the PLM axon. Like the existing *wdfy-3(ok912)* allele, the new *wdfy-3(cue30)* null allele can suppress PLM axon termination defects caused by an *fsn-1* null allele. Creating and characterizing new *wdfy-3* alleles will increase our understanding of this gene and could help elucidate more of the gene’s conserved functions.

**Figure 1. A new deletion allele of wdfy-3 causes mild axon termination defects and suppresses axon termination defects caused by an fsn-1 null allele.**
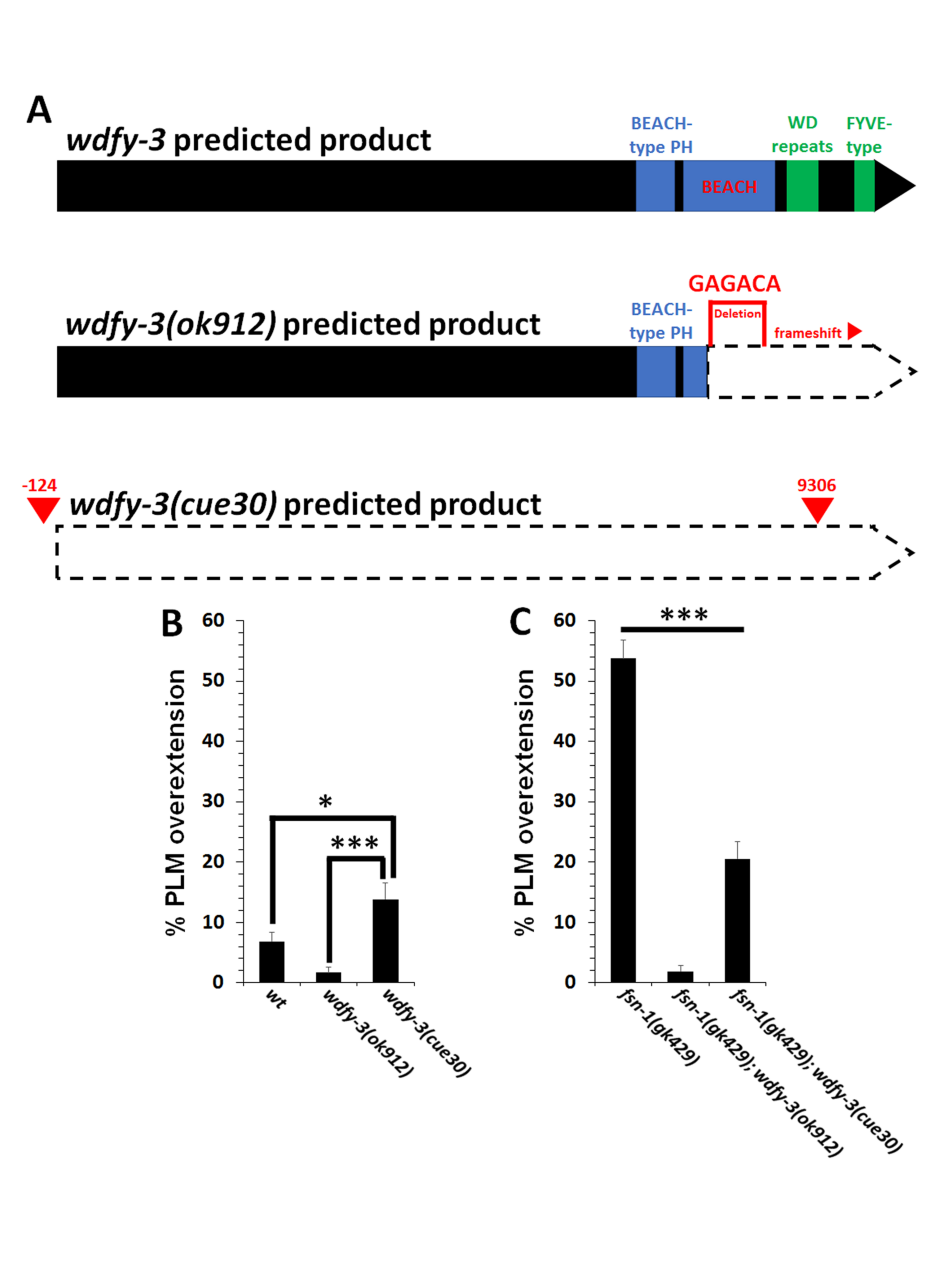
**(A)** Schematic of the predicted protein products for wildtype *wdfy-3* , *wdfy-3(ok912)* and *wdfy-3(cue30).* The *wdfy-3(ok912)* allele consists of a small deletion (marked by red bracket), insertion of GAGACA and resulting frameshift. Therefore, the BEACH, WD repeats, and FYVE-type domains are predicted to be non-functional in the protein product of the *wdfy-3(ok912)* mutant. The newly created *wdfy-3(cue30)* removes the first 33 of 36 exons and is predicted to be a null allele. Red arrows mark the cut sites of Cas9, with the base pair number relative to the start codon indicated above the cut site. **(B)** Quantification of PLM axon overextension defects in wildtype (wt), *wdfy-3(ok912)* and *wdfy-3(cue30)* animals. The null *wdfy-3(cue30)* animals showed increased penetrance of PLM axon overextension defects relative to wildtype and *wdfy-3(ok912).* **(C)** PLM axon overextension defects caused by the *fsn-1(gk429)* null mutation are suppressed by the *wdfy-3(cue30)* and *wdfy-3(ok912)* mutations. Axons were visualized with the *muIs32* transgene that encodes *Pmec-7::gfp* . Between 150 and 400 axons were observed in L4 stage hermaphrodites per genotype. Asterisks indicate statistically significant difference, Z-test for proportions (*p<0.01; ***p<0.0001). Error bars represent the standard error of the proportion.

## Description

The human *WDFY3* gene (also known as Alfy) encodes a scaffold protein that is required for selective autophagy and has been associated with autism (Clausen et al., 2010; De Rubeis et al., 2014; Filimonenko et al., 2010; Iossifov et al., 2012; Yuen et al., 2017; Wang et al., 2016). WDFY3 functions as an adaptor that links ubiquitinated cargo to the core autophagy components, thereby recruiting ubiquitinated targets into autophagosomes for eventual destruction in autophagolysosomes. Large scale genome sequencing studies have revealed a significant association between *WDFY3* and autism (De Rubeis et al., 2014; Iossifov et al., 2012; Yuen et al., 2017; Wang et al., 2016). Consistent with this observation, *WDFY3* is required for the normal development of axon tracts in mice (Dragich et al., 2016). Moreover, genetic analysis in *C. elegans* has indicated that *wdfy-3* regulates axon targeting by functioning in a genetic pathway with *egl-19* , an ortholog of the *CACNA1C* autism-associated gene (Buddell et al., 2019). However, the mechanism through which WDFY-3 affects axon targeting remains mostly unknown.

As a first step in further exploring the role of WDFY-3 in axon targeting, we used CRISPR Cas9 to create the new *wdfy-3(cue30)* allele. This new *wdfy-3(cue30)* allele is a near complete deletion of the coding region of *wdfy-3,* except for the last three exons that make up the FYVE-type domain at the C-terminus of the protein (Figure 1A). This is unlike the previously studied loss-of-function *wdfy-3(ok912)* allele, which contains a small deletion that removes part of the BEACH domain in the C-terminal region (Figure 1A). The removal of the BEACH domain in the *wdfy-3(ok912)* allele also causes a frameshift mutation, likely resulting in the disruption of the WD repeats and FYVE-type domains (Kinchen et al., 2008). However, the large N-terminal region of WDFY-3 may not be affected by this mutation.

To determine how our new *wdfy-3(cue30)* allele affects axon targeting, we examined the PLM axon. The PLM cell body resides in the tail and extends an axon anteriorly along the lateral body wall. In wildtype worms, the PLM axon terminates at the midbody, prior to reaching the ALM cell body (Chalfie et al., 1985). We found that our new *wdfy-3* deletion allele causes PLM axon termination defects that are significantly greater than those observed in wildtype (Figure 1B). Interestingly, the *wdfy-3(ok912)* allele does not cause PLM axon termination defects. Together, these observations suggest the possibility that the *wdfy-3(ok912)* allele might not be a null allele and that the uncharacterized N-terminal region of WDFY-3 could have functions important for axon guidance. Future investigations could test this hypothesis through the analysis of additional targeted deletions in *wdfy-3* .

Our prior work demonstrated that the *wdfy-3(ok912)* allele can suppress axon termination defects caused by an *fsn-1* null allele (Buddell et al., 2019). To determine if our new *wdfy-3(cue30)* allele also has this property, we analyzed PLM axon termination in *wdfy-3(cue30)* ; *fsn-1(null)* double mutants. We found that *wdfy-3(cue30)* also suppresses overextension caused by *fsn-1* (Figure 1C). However, this suppression did not bring the overextension penetrance back to wildtype levels like the *wdfy-3(ok912)* allele (Figure 1C). These observations are consistent with our previous report of a genetic interaction between *wdfy-3* and *fsn-1* (Buddell et al., 2019).

## Methods

A CRISPR editing technique was used to create the *wdfy-3(cue-30)* mutation. Unique guide RNAs were designed to cut in two places to excise most of the coding region of the *wdfy-3* gene without perturbing the function of nearby genes or the non-coding UTR region of *wdfy-3* . Guide RNAs used were upstream: GCGATTATTGGATTATCTCG & downstream: AGTTTTGAACACGTGCGACG.

*C. elegans* strains were cultured and maintained on nematode growth medium (NGM)-agar plates using standard methods at 20°C (Brenner, 1974). Axons were labeled and observed as previously described (Xu & Quinn, 2012). Briefly, animals were mounted on a 5% agarose pad and observed with a 40x objective. PLM neurons were visualized with the *muIs32* transgene which encodes *Pmec-7::gfp* + *lin-15(+)* and is expressed in all mechanosensory neurons (Ch'ng et al., 2003).

## Reagents

AGC52: *muIs32* [ *mec-7p::gfp* + *lin-15* (+)] II; *fsn-1(gk429)* III

AGC135: *muIs32* [ *mec-7p::gfp* + *lin-15* (+)] II; *wdfy-3(ok912)* II

AGC209: *muIs32* [ *mec-7p::gfp* + *lin-15* (+)] II; *wdfy-3(cue30)* IV

AGC259: *muIs32* [ *mec-7p::gfp* + *lin-15* (+)] II; *fsn-1(gk429)* III *; wdfy-3(ok912)* IV

AGC260: *muIs32* [ *mec-7p::gfp* + *lin-15* (+)] II; *fsn-1(gk429)* III *; wdfy-3(cue30)* IV
